# Activity Monitors as Support for Older Persons’ Physical Activity in Daily Life: Qualitative Study of the Users’ Experiences

**DOI:** 10.2196/mhealth.8345

**Published:** 2018-02-01

**Authors:** Maria Ehn, Lennie Carlén Eriksson, Nina Åkerberg, Ann-Christin Johansson

**Affiliations:** ^1^ School of Innovation, Design and Engineering Mälardalen University Västerås Sweden; ^2^ Västerås Municipality Care and Welfare Västerås Municipality Västerås Sweden; ^3^ School of Health, Care and Social Welfare Mälardalen University Västerås Sweden

**Keywords:** exercise, behavior, aged, seniors, mobile applications, fitness trackers

## Abstract

**Background:**

Falls are a major threat to the health and independence of seniors. Regular physical activity (PA) can prevent 40% of all fall injuries. The challenge is to motivate and support seniors to be physically active. Persuasive systems can constitute valuable support for persons aiming at establishing and maintaining healthy habits. However, these systems need to support effective behavior change techniques (BCTs) for increasing older adults’ PA and meet the senior users’ requirements and preferences. Therefore, involving users as codesigners of new systems can be fruitful. Prestudies of the user’s experience with similar solutions can facilitate future user-centered design of novel persuasive systems.

**Objective:**

The aim of this study was to investigate how seniors experience using activity monitors (AMs) as support for PA in daily life. The addressed research questions are as follows: (1) What are the overall experiences of senior persons, of different age and balance function, in using wearable AMs in daily life?; (2) Which aspects did the users perceive relevant to make the measurements as meaningful and useful in the long-term perspective?; and (3) What needs and requirements did the users perceive as more relevant for the activity monitors to be useful in a long-term perspective?

**Methods:**

This qualitative interview study included 8 community-dwelling older adults (median age: 83 years). The participants’ experiences in using two commercial AMs together with tablet-based apps for 9 days were investigated. Activity diaries during the usage and interviews after the usage were exploited to gather user experience. Comments in diaries were summarized, and interviews were analyzed by inductive content analysis.

**Results:**

The users (n=8) perceived that, by using the AMs, their awareness of own PA had increased. However, the AMs’ impact on the users’ motivation for PA and activity behavior varied between participants. The diaries showed that self-estimated physical effort varied between participants and varied for each individual over time. Additionally, participants reported different types of accomplished activities; talking walks was most frequently reported. To be meaningful, measurements need to provide the user with a reliable receipt of whether his or her current activity behavior is sufficient for reaching an activity goal. Moreover, praise when reaching a goal was described as motivating feedback. To be useful, the devices must be easy to handle. In this study, the users perceived wearables as easy to handle, whereas tablets were perceived difficult to maneuver. Users reported in the diaries that the devices had been functional 78% (58/74) of the total test days.

**Conclusions:**

Activity monitors can be valuable for supporting seniors’ PA. However, the potential of the solutions for a broader group of seniors can significantly be increased. Areas of improvement include reliability, usability, and content supporting effective BCTs with respect to increasing older adults’ PA.

## Introduction

### Background

Physical activity (PA) has numerous health benefits in all age groups. For older persons, it can contribute to maintenance of autonomy and quality of life. Older adults value their independence, but health-related consequences from fall injuries pose an immediate threat to their ability to remain self-sufficient. Hence, falls are a major health concern, which needs to be prevented in the old population. Moreover, successful fall prevention can reduce large economic costs for the society.

There exists evidence that 40% of all fall injuries can be prevented by regular PA [1]. For this purpose, exercise programs including training of balance, muscle strength, endurance, and aerobic exercises are recommended [2,3]. In addition, general PA, which can be defined as “any bodily m ovement produced by skeletal muscles that results in energy expenditure above the basal resting level” [4] can delay functional decline and reduce the risk of premature mortality of the old population. Walking activities are major contributors to general PA among healthy older adults [5]. Compliance to exercise programs is generally low in the old population; Riebe and Burbank report a 30% decrease of exercise activities only 4 weeks after an exercise program was introduced [6]. Different approaches have been tried to increase the adherence of older adults to exercise programs: technology-based interventions (mainly with commercially available gaming technology) have shown promising results in terms of adherence at least throughout the first 12 weeks of the intervention [7]. However, to increase long-term exercise compliance and also general physical activity, a behavior change process is required; in this process support from caregivers are decisive [8]. Here, different types of technical support systems can be of value [9,10].

### Prior Work

Persuasive technology is designed to change people’s attitudes and behaviors [11]. Persuasive systems have been used in health care to increase patients’ adherence to Web-based interventions [12] and to promote PA [13,14]. Most likely, this type of systems can be useful for promoting seniors’ PA contributing to fall prevention. However, the systems need to support behavior change techniques (BCTs) effective for the specific target behavior and intended user group [15]. Furthermore, the systems must meet users’ needs and preferences. Here, aspects critical for usability [16] and user acceptance [17] are important to gather.

Commercial activity monitors (AMs) are examples of persuasive technology for increasing people’s PA [18-20]. However, the available AM products have proven to be insufficient for monitoring PA of older adults with reduced walking speed and with varying gait pattern [21]. Moreover, BCTs supported by current AMs (mainly self-monitoring and self-regulation techniques) have proven as less efficient for supporting behavioral change among older adults than for younger adults [22,23]. It has been suggested that wearable AMs should be enriched with additional BCTs that are specifically efficient for increasing older adults’ PA [23]. Examples of such BCTs are “provide rewards contingent on successful behavior,” “barrier identification or problem solving,” and “model or demonstrate the behavior.” Recently, a quantitative investigation of older peoples’ experiences with commercially available AMs for self-tracking PA behavior was performed in terms of drivers technology use according to the Technology Acceptance Model (TAM) [24,25]. However, a review of empirical research on technology acceptance by older people concludes that, to better understand older people’s acceptance behavior, additional variables should be included in TAM [26]. Qualitative studies of older person’s experiences in using AMs are important for understanding users’ acceptance of the technology. A mixed-methods study has assessed the acceptance and usage of wearable activity trackers among Canadian community-dwelling adults in the age range of 55 to 84 years [27]. Previous studies have investigated the acceptance of AMs among persons with chronic illness [28] and the usability of AMs among patients with chronic obstructive pulmonary disease [29].

To prepare for user-centered design [30] of new solutions supporting seniors in increasing their PA behavior, we investigated how a group of persons in the age range of 75 to 90 years experienced using currently available AMs in daily life. Our intention was to perform the study in a setting very similar to the real life of a senior that has acquired an AM and tries to use it as support for daily PA.

The aim of this study was to explore senior users’ experiences in using the current AMs and from that learn more about users’ requirements and preferences related to motivation, meaningfulness, usefulness, and usability.

## Methods

**Figure 1:**
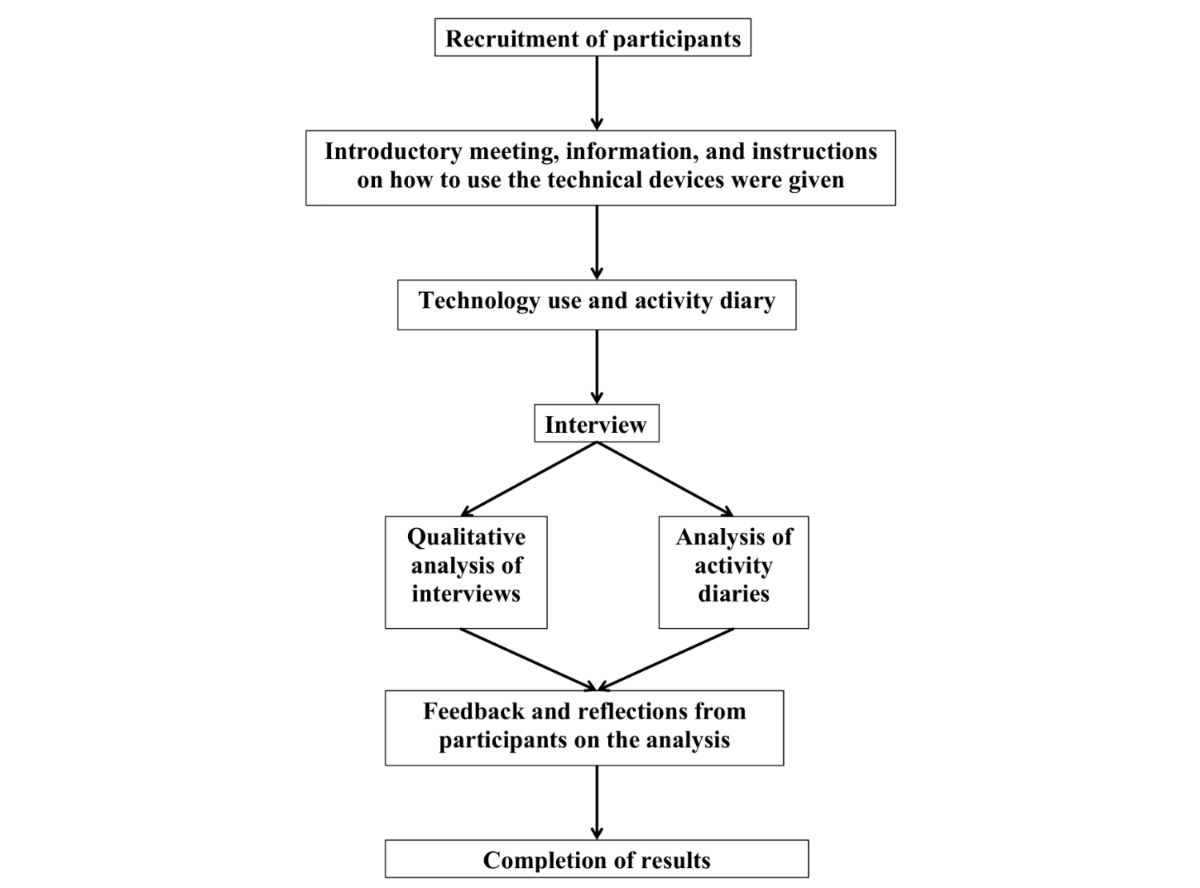
Overview of the applied study design.

An overview of the applied study design is presented in Figure 1.

### Study Design

The study was descriptive with a qualitative inductive approach [31] to gain understanding of older persons’ experiences in using for measuring their daily PA in terms of steps per day.

Data on users’ experiences have been collected from different sources including interviews, activity diaries, and documentation of group discussions with the participants on the analysis result.

### Participants and Recruitment

A total of 8 participants, 75 years or older were recruited, of which 6 had recently finished participating in a study in which exercises to prevent falls had been evaluated [32]. Two participants responded positive to participation via an advertisement in a meeting place for old people in the community. Six of the participants had light walking disabilities and used walkers on wheels, and 2 participants walked without aid.

All 8 individuals who were asked for participation responded positively, and written consent was collected from them.

Inclusion criteria included being 75 years or older and living in an ordinary home in the community. Exclusion criteria included not being able to move independently at home and cognitive disability, both of which were considered as threats to validity for experience evaluations. Participants who had finished the previous exercise study had all a score of 25 or more at the Mini Mental State Examination [33]. They were all tested during the latest year, and experienced physiotherapists in the field judged the participants who responded to the advertisement as having sufficient cognitive function.

### Activity Monitors and Tablet-Based Apps

Two commercially available bracelets for monitoring PA were used in the tests, namely Withings Activité Pop (Withings) and Jawbone UP3 (Jawbone), together with corresponding software (apps) accessible on a tablet (iPad, Apple). The devices (wearables and iPad) were selected as they were considered to be user-friendly, hygienic, and enabled storing activity data only locally on the tablet. To keep data locally, social features of the solutions were not enabled and therefore, not used. Moreover, the inclusion of two different products makes our results representative for more than one specific AM.

The Withings wearable has the design of an analog wristwatch with a major dial displaying current time and a smaller dial giving real-time feedback in terms of percentage of daily activity goal achieved. The Jawbone wearable is designed as a bracelet with three icons (status light) that can be lit up. Different kinds of notifications can be given to the users on the band. In this study, the Jawbone bracelet was used for monitoring purposes, and users were instructed to access activity results in the app.

Both wearables monitor PA and sleep cycles. In addition, the Jawbone bracelet continuously monitors resting pulse. As a consequence of this, the battery of the Jawbone bracelet needs to be charged every 3 to 4 days, whereas the battery of the Withings bracelet lasts for 8 months.

Each wearable is packaged with a specific app to be used on a tablet or a smartphone.

The Withings app gives the user an overview of daily activity data, both current and historical. Data shown include total amount of steps taken (absolute number and percentage of the daily activity goal) and steps taken per hour over the day visualized in a bar diagram. If sleep has been measured, total hours of sleep, percentage of sleeping goal, and a graph showing sleeping activity per night hour is also shown. Moreover, if specific activities (such as running and swimming) have been identified, a corresponding summary of the measured activity is shown (duration, energy consumption, and if applicable, also distance). If the user receives badges as rewards for healthy days (eg, if the activity goal had been reached), this is also shown in the summary. The start page of the Jawbone app shows a daily overview of accomplished PA (steps taken) and total sleep time. Furthermore, the user can get feedback on trends of different behavior over longer times. In addition, the user can receive feedback on measurements related to PA (including total amount of steps, % of activity goal and total active time, longest active period, and longest active idle period in terms of duration and energy consumption) and resting pulse. Sleep analysis is also summarized and visualized. The app allows the user to set quantitative goals for target behaviors including steps per day and sleep hours per night. General recommendations for each goal are given.

### Intervention

Two participants came to the university to meet physiotherapists and to receive the technical devices, additional information, and instructions. At this occasion, the participants responded to some questions regarding short personal information, general health, and activity habits. The participants received thorough oral and written information about the technology and were introduced to handling the devices and charging the batteries. Questions and comments were encouraged to elucidate unclear information and doubts in relation to the devices. All participants tested the use of both the bracelets and the iPad on this occasion. The participants borrowed the devices and started to use them the following day. In addition, the participants received an activity diary in which they were requested to estimate their physical effort each day during the test period. Here, the participants could also note additional information about experienced difficulties with the technology and activities performed.

Participants were instructed to pursue daily activities as usual, wear the activity bracelet all day and preferably also at night, fill information in the activity diary on estimated physical effort daily, and whether the technology had been functional. Moreover, participants were instructed to open the app on the tablet once a day to look at the results from their registered activity.

Six participants were in the same manner informed at their home by the physiotherapist that they had been in contact with during the previously finished study [32]. They also borrowed the devices, were requested to fill in the activity diary, and started to use the devices the day after the visit.

During the test period, each physiotherapist kept in contact with her participants to check if the testing went on well and if the technology was OK. The participants also had the opportunity to call the physiotherapists for support during the test period, if needed. The participants tested the technology for 9 to 10 days.

### Data Collection

#### Background Characteristics

Age, gender, length, general health, medications, use of walking aid, help in daily life, perceived memory capacity, and PA level were collected through a questionnaire at the initial meeting. Participants estimated their PA level by using the five-level scale that is frequently used by the Public Health Agency of Sweden. It is further developed into a compatible seven-level scale, which is recently validated with a correlation coefficient of .7 with AMs [34]. The participants’ previous experiences from using mobile phones, tablets, phone-based pedometers, and computers, respectively, were collected in the interviews.

#### Activity Measurements

During the test period, activity was monitored and data for each participant was saved in the corresponding app on the tablet. Data from each participant (mainly in terms of number of steps/day, in some cases also sleep hours and activities identified by the technology) was moved from the tablet to a local data server.

#### Activity Diary

Each participant was asked to self-report in a diary both daily physical effort by giving a score on a scale from 0 to 10 (where 0=no effort at all and 10=maximum effort) in a diary and technology functioning feedback (yes or no). Participants were also asked to report descriptions of activities performed and experienced problems with the technology. Moreover, when reporting technology malfunctions, participants were asked to describe what kind of problem they had experienced.

#### Interviews

Individual semistructured interviews were conducted by one of the authors (ACJ). Each interview lasted for approximately 30 min. The participants were preliminary informed that the purpose of the interview was to explore their experiences with the technical devices and to share their experience of using the technology in their daily lives. A semistructured interview guide [35] was used, containing five main questions supported by follow-up questions to initiate reflections and to obtain descriptions of the experience of using the technical devices. The interview guide is presented in Multimedia Appendix 1. The interviews were audiorecorded and transcribed verbatim.

#### Meeting With the Users

The participants were invited to a meeting where the results from the interviews and the technical measurements were presented by the researchers. At this meeting the participants were also encouraged to make reflections, give feedback, and completions of presented results. Group discussions at the meeting were documented.

### Data Analyses

#### Background Characteristics

The quantitative data related to the participants’ background information were analyzed by means of descriptive statistics.

#### Activity Diary

The quantitative data self-reported in the activity diaries were analyzed through descriptive statistics. These data included scored daily physical effort and daily report on the technology functioning. Qualitative data in the diaries included comments on experienced problems with the technology and examples of performed activities. These data were summarized for the study group.

#### Interviews

A qualitative content analysis was conducted by adopting an inductive approach [31]. Throughout the analysis, categories and subcategories were generated from the interview text. The analysis began with ACJ reading all transcripts, thoroughly several times. Next, to capture the key concepts and thoughts, the text was read word by word to extract meaning units, with a focus on the experiences of measuring PA with the adopted technical devices. The text was condensed into meaning units by ACJ and ME and subsequently coded by all authors. Codes were discussed and finally set in agreement with all authors; the codes emerged from the content of condensed meaning units. The coded meaning units were grouped into categories based on similarities in the content and subcategories, which reflected different aspects of the content. A coding scheme was used. Finally, the analysis resulted in descriptions of three categories with 13 related subcategories. During the analysis process, all authors discussed units, codes, and categories until agreement was reached. All authors followed every step in the analysis, confirmed, or raised questions, which needed to be discussed. The final version of the analysis was read by all authors to ensure the rigor of the described categories and subcategories [36]. In addition, quotations were used to illustrate the text and to give examples from the interviews, with the aim of achieving trustworthiness.

In striving for trustworthiness and credibility, reflexivity was used in the analysis. The authors strived to become aware of their preunderstandings how those might influence the emerging findings toward how the categories covered the data. The categories were thoroughly discussed to elicit differences between and similarities within the categories. In striving for credibility, methods the selection of participants, data collection, and data analysis are presented as thoroughly as possible. All authors were aware of the preunderstanding and existing knowledge about the context. The researcher ACJ performing the interviews was also aware of the physiotherapist lens, which she possessed.

#### Meeting With the Users

Notes from the meeting were read by all authors and analyzed in comparison to the interviews. Analysis was performed through group discussions between the authors.

### Ethics

The study was approved by the regional ethics committee in Uppsala (Dnr 2015/372). All participants were given both verbal and written information about the study; then, informed consent was obtained from all participants.

## Results

### Participants

Eight eligible participants, all in independent living, were included according to the inclusion and exclusion criteria. Their mean age was 83 years (range 77-90 years), and the mean of medications used per person was 3 (range 1-8). Descriptive statistics from background information of participants are presented in Table 1.

All participants were familiar to and used mobile phones, no one had previously used an iPad, and two had used an app-based pedometer on a smartphone. Two participants were familiar to and used computers, mainly for mail correspondence.

All participants completed the testing period. However, one participant became sick during the test period and therefore, only wore the AM and filled in the notebook during 5 days.

### Activity Measurements

All the participants performed activity measurements during the whole testing period; however, one person became sick and 3 participants experienced technical problems during the tests. As a consequence, PA information (monitored by AMs) about those four persons is missing for several days in the test period. However, all participants wore the monitors and filled in information in the diaries during the whole period.

As the aim of the study was to investigate the users’ experiences in using AMs, results from the activity measurements have only been used for comparison with self-reported data in the diaries. Moreover, the amount of measurement data was very limited. Therefore, the AM results are not presented in the paper. Comparisons of AM data and the activity diaries indicated that the measurement correspond to the self-reported daily physical effort to a certain extent. Moreover, the comparisons indicate that reliability of measurements related to some types of physical activities such as biking and walking with a walker need to be further explored. Indeed, in the interviews, participants raised questions and comments regarding the reliability of measurements for these types of activities.

### Activity Diary

All participants completed diaries. One participant was sick during the testing period and therefore, only reported 5 days.

Self-estimated daily physical effort was in mean score 4 (standard deviation 2, range 1-9). Mean score per day for each participant varied between 2.2 and 7.1. Activities reported in the diaries included walks, biking, gym training, shopping, and cooking, and one participant had extra work serving in a café.

Out of 74 total test days, participants had perceived that the technology had been working 58 days (78%); nonworking 14 days (19%); 2 days (3%) lacked this information. Problems reported were difficulties in getting the app window in the right orientation (was now upside down), failure in charging the bracelet (only Jawbone), lost Bluetooth connection between bracelet and tablet, difficulties in finding training results in the app, and unwanted popping-up of text messages on the tablet. In several cases, participants reported nonworking technology without adding further details describing how. One participant also described to be insecure about whether the technology had been functional, as previous experience in using computers was very limited.

**Table 1:** Descriptives of the participants.

Participant characteristic		Total, n (%)
**Age (years)**		
	75-80	3
	80-85	3
	85-90	2
**Gender**		
	Female	6
	Male	2
**Use of walking aid**		
	No	3
	Only outdoors	3
	Both indoors and outdoors	2
**Use of medications**		
	1-4 medications	4
	5-8 medications	4
**Weekly amount of activity causing increased body temperature**		
	>5 hours/week	3
	3-5 hours/week	3
	1-3 hours/week	1
	Missing data	1
**Physical activity performed over the last 6 months**		
	2-4 hours/week of lighter physical effort	7
	>3 hours/week of more intense physical activity	1
**Experiences of using technical platforms**		
	Mobile phones exclusively for making calls	8 (100)
	Computers	2 (25)
	Tablet	0 (0)

### Interviews

The participants’ overall experiences in using the monitors were investigated through qualitative analysis of the whole interviews. Three main categories and 13 subcategories emerged from the interviews. Main and subcategories are presented in Multimedia Appendix 2 (Main and subcategories based on the interviews).

#### Influence on the Individual

The participants expressed that the activity monitors had, to varied extent, influenced their motivation, awareness, emotions, and behavior related to daily PA.

The degree to which the users’ motivation for PA had increased when using the monitors varied in the group. Some users saw that the monitors had motivated them to be more physically active and encouraged PA:

I was motivated by the technology, that I freely admit.

On the other hand, other participants described that they were already motivated for PA, and this was not changed because of the monitors:

The technology has no impact on my motivation, I am physically active anyway. I am on the verge to getting diabetes, that is what motivates me the most.

It was also pointed out that using the monitors requires a basic degree of motivation for PA and interest in progress:

If you are interested in making progress with exercises and things like that, it is good (the technical support). But for those who are not really motivated, it is a matter of motivating people.

Furthermore, using the monitors and apps provoked different emotions among the participants: some participants found it enjoyable and interesting to measure and get feedback on performed physical activities. Hence, PA was perceived funnier when the user could see how active he or she had been, for example when an activity goal had been reached:

You could see how far you have walked, I have not registered that previously. It was fun.

Different results of the activity measurements could cause different kinds of feelings among the users, for example, low activity feedback could cause feelings of embarrassment:

It was irritating when it is visible that I had been so damn lazy. But it is good to have (the technology).

Likewise, the user could be positively surprised if the measured activity was higher than expected:

Yes, I was positively surprised over that I had taken so many steps. I hadn’t walked that much (laugh). I was positively surprised.

Negative feelings were also provoked in situations when the devices failed to work as expected:

I was disappointed when it stopped working.

Participants described that their awareness of how active they actually were had increased because of using the monitors: the measurements clearly reflected whether the user had been active or inactive during the day. Furthermore, users perceived interests in comparing measurement results in terms of steps from different activities. Here, some participants were surprised to see that also indoor activity could lead to high number of registered steps:

I was surprised that I got the highest number of steps during the day that I spent indoors. But I was active 8-9 hours in a row. Out buying cream, in again, up and down.

I found it interesting to see the results from different activities performed. The difference between an active and inactive day was clear.

Some users experienced that the activity measurements had an effect on their PA behavior in the sense that they increased their PA:

I have walked a little more while being monitored.

The participants emphasized the impact of the monitors as reminders and a push forward to increase PA. They described that feedback in terms of reminders was important for behavioral change toward a more active life style. Additionally, goal setting was perceived important for increasing active behavior: a quantitative activity goal was helpful for the user by clarifying if the current activity level was too low. In addition, reminders about the goal could stimulate the user to increase and maintain activity:

Setting goals has importance, I get pushed if I have been too lazy.

Some participants described that they were already active to a certain level in their daily lives. They had their own considerations, decisions, and habits related to PA, and those were not affected by measuring the activity:

I did not change my exercise habits during the monitoring, I took the same walk as usual in the morning or in the afternoon. It is a goal I have and as a pensioner, I have plenty of time.

#### Experiences From Being Monitored

The participants expressed their experiences from being monitored in terms of limitations, possibilities, integrity, reliability, and feedback.

Some participants envisioned that the activity monitors and apps might have a limitation in their usability and usefulness for senior persons: Participants saw that, for senior persons less vigorous than themselves, everyday use of the devices could be difficult, cumbersome, and demanding:

It is more difficult for a person less alert than me maybe also using walking aids. It might be tough for them to register like this every day.

Furthermore, the usefulness of the monitors and apps could also be limited to persons with certain attitudes and mentality.

Some users had perceived the devices as fragile and had therefore limited their use and own experimentation with the technology to avoid destroying it. For example, some persons had abandoned opening the app on the tablet for studying activity results.

Furthermore, technical limitations of the devices were described: the users highlighted that tested monitors were limited in their capability of measuring different kinds of activities. For example, gym and household activities such as baking had not been registered. This was disappointing for persons that had performed these activities.

The possibility of increasing the users’ PA by means of the monitors was discussed. In particular, feedback on current activity in relation to a goal was seen helpful and enabling the user’s self-control: by increasing the user’s awareness on whether current activity behavior is sufficient, the person can be stimulated to increase his or her PA. Additionally, the feedback might encourage and promote the user to increase PA. However, the participant describing this possibility was at the same time expressing doubts on how efficient the devices would be in this aspect:

I think it would spur others that don’t move so much. Because he or she would then need to present something. And that I think can be a real spur. So for many people it will probably be a spur because I don’t want to appear worse than others.

Another enhancement possibility of the monitoring technology proposed by the participants was the combination of PA measurements and health parameters (such as pulse and blood pressure). Moreover, the users discussed improvement possibilities for the tested monitors. For example, the Jawbone bracelet could be redesigned to better instruct and facilitate charging:

There should have been an instruction saying “Check charging here” and a symbol on the bracelet that could be clicked on in order to see the charging level.

In general, participants perceived no problems concerning integrity associated with having their personal PA measured by the monitors. However, it was envisioned that other persons might feel controlled if being monitored:

Someone really sensitive in terms of integrity might feel controlled, but for me it is only positive.

Furthermore, one participant described that she felt afraid of being pushed into something she had not decided herself:

I want to decide myself how many steps I should take. I don’t think I need a specific goal.

One important aspect of users’ experience related to the measurements was reliability, both of the measurements, and the devices, Problems with the devices negatively affected the participants’ motivation in continuing with their use:

I wore the bracelet during the first night but when problems began to occur, I didn’t bother using it at night.

The users’ perception of the measurements reliability was highly dependent on how well the measurement results correlated with the individual’s own estimation of activity level. In fact, some participants suspected that the measurements had failed to work properly and questioned whether the result was correct. This reduced their motivation for being monitored.

User experiences from getting feedback on accomplished activity in terms of steps taken varied among participants. Although some participants questioned the importance of feedback, others were positive:

It would feel great, because it is what you need. You need the push that you should walk.

The participants reflected on what type of feedback might be most helpful for them to increase their PA: seeing the activity results was perceived interesting and appreciated in terms of receipt confirming how active one has actually been. Especially, the feedback should clearly confirm the user whether a daily activity goal had been reached or not:

I think the idea is great. At least for me because I want confirmation of my outdoor walks. So it was actually perfect.

Moreover, the importance of praise in terms of feedback was emphasized—even in cases when the progress was modest.

Participants perceived that the measurements enabled self-monitoring. However, all participants expressed low interest in seeing their accomplished PA on a screen. It was pointed out that software providing feedback must be very easy to use and navigate in. For example commands must be in the user’s native language.

#### Experiences in Using the Technical Devices

Users’ experiences in using the technical devices were mainly related to handling, insecurity, learning, and wearing the monitors.

In general, the users perceived handling the monitors easy. However, the users described that they had felt insecure on whether the communication between the monitor and app would work. Moreover, participants had felt insecure on whether the monitors could be damaged if worn while taking a shower. Furthermore, the Jawbone users had felt insecure on whether they had handled the charging of the bracelet correctly:

I was of course a bit worried initially about not being able to handle it. That I would push the wrong button and things like that. But then I thought it worked as the physiotherapist had taught me and I tried to remember that. Yes, it has worked well. I think.

Handling the tablet could cause frustration and insecurity: the participants felt insecure on how to interact with the touch screen as they lacked previous experience of swiping hand or fingers over the screen and found the movement being difficult to perform. The participants had felt inexperienced in handling the technical devices and therefore had felt insecure on whether they were doing this correctly. In addition, there were occasions when the technology had not worked properly, and this made the users wonder if the problems experienced were because of incorrect handling or to technical failure:

I wish I would be because it is really good to know these things. Without knowledge, help is needed for everything. If you want...if you can manage a personal computer, you are able to proceed directly. So I wish, and if I had known more, then this would have...then I would have felt more confident and then it would have worked although I feel a bit hesitant.

The users expressed a desire to learn more about how to use the technical devices. Moreover, they would have preferred increased access to help in the early phases of learning the practical handling. Here, some participants had experimented on their own to learn how to handle the technology. Meanwhile, others had refrained from doing this as they were afraid of damaging something:

If I would change anything, it would be that I should have learnt more so that I had felt more confident in the beginning.

The written instructions provided to the participants contained English terms. As the participants have another native language, learning and following the written instructions was perceived difficult:

Then I read the written instructions but they contained a lot of English, there shouldn’t be English terms there.

The wearable bracelets were in general perceived user-friendly, unobtrusive, and easy to wear. However, the Jawbone bracelet was perceived stiff and the Withings watch was found large, uncomfortable, and difficult to match with different types of clothes:

...of course it was large and awkward sometimes. When wanting dress nicely, it was of course not so neat.

Furthermore, the watch was described as difficult to put on, and help from another person had been needed to lock the wristband:

I once took off the watch while taking a shower, but after that I have worn it during showers because it was very difficult to put on. It was hard to hook, I had to take help.

The participants wore the monitors during daytime. Some users also wore the bracelets at night while others took them off at night, mainly as they found the wearables uncomfortable or were used to sleeping without watch. Although the participants had been instructed to wear the monitors during showers, several users took them off while showering. Furthermore, users who had worn the bracelets in the shower described that they had still somehow been careful not to get the device wet:

I have worn the watch in the shower but have been careful so that it wouldn’t get too wet. I was a little careful with it.

### Meeting With the Users

All 8 participants were invited to a meeting with the research group where results from the data analysis were presented and discussed. Five participants could not attend because of various reasons; 3 participants came to the meeting. The results from the interviews were presented and discussed.

In discussions on being monitored, the participants confirmed that they had been surprised when seeing that outdoor activities performed with walker resulted in fewer steps than indoor activities without walker. There were also participants that had continued measuring PA after the study by using a mobile phone.

In discussions on handling the technology, participants emphasized that the technology must be easy to handle. In fact, it was expressed that the technology has to be “so user-friendly that the user doesn´t even perceive it as technology.” Furthermore, the participants emphasized the importance of access to practical training on how to handle the technology together with another person.

In discussions on feedback from the technology, the participants expressed the desire of being informed in case they had moved too little. Feedback on insufficient activity should preferably be presented directly on the wearable monitor so that no extra screen is needed. Participants also described that it would be interesting to monitor and get feedback on different health parameters. Additionally, information on available basic fall preventive exercises was found valuable.

Different aspects of the technology’s quality were discussed: the participants pointed out usability and intelligibility as most important. Access to personal support was also described as highly important, especially for long-term use.

## Discussion

### Principal Findings

This study has provided insight on how community-dwelling older adults experienced using commercial activity monitors for a relatively short time period with limited access to help and support. The study setting is comparable to the real-world situation of senior citizens acquiring a commercially available support for PA in daily life. More specifically, the addressed research questions were as follows:

- What are the overall experiences of senior persons, of different age and balance function, in using wearable AMs in daily life?
- Which aspects did the users perceive relevant to make the measurements as meaningful and useful in the long-term perspective?
- What needs and requirements did the users perceive as more relevant for the activity monitors to be useful in a long-term perspective?

PA has many health benefits for the increasing old population [37]. However, a major challenge is to achieve and maintain increased PA among older adults. Support for behavioral change can contribute here. Persuasive technology is designed to support behavioral change including increasing PA [11,38]. Current products for promoting PA are well adopted in the younger population but are not in their current state suitable for the old population [18,27,39]. In our study, the activity measurements terminated because of technical problems for 3 out of 8 participants. Although the study sample was small, this indicates that current AMs can be challenging to handle for senior users.

To design new persuasive systems, the users’ needs and preferences must be understood, for example, regarding motivation and usability [16]. This is often obtained by codesigning new solutions in cooperation with end users [30]. The activity monitors in the study support several BCTs including goal setting, discrepancy between current behavior and goal, feedback on behavior, and self-monitoring of target behavior [9]. Although these BCTs have been shown effective for increasing younger adults PA, their effectiveness for increasing older adults PA has been questioned: for example, a systematic review [22] has identified three BCTs (namely “provide rewards contingent on successful behavior,” “barrier identification or problem solving,” and “model or demonstrate the behavior”) as significantly effective for increasing older adults’ PA. Due to the qualitative approach of our study in combination with a limited sample and short intervention time, no conclusions can be drawn from our results regarding the efficiency of BCTs supported by the monitors for this user group. However, the qualitative investigation of the users’ experiences in our study has enabled us to identify aspects important for the measurements’ meaningfulness and usefulness, as well as needs and requirements of the supporting technology.

The users’ descriptions about how the measurements had influenced their motivation, awareness, emotions, and behavior illustrated that the monitoring of PA had increased their awareness on own PA behavior. In addition, users had started to explore and reason about how many steps each of the different activities could correspond to. As a consequence, PA could become funnier and some persons had increased their PA. Persons reaching their PA goal experienced feelings of enjoyment, whereas other people felt embarrassed for having low PA. Hence, measurements were perceived meaningful and useful for the users as they provided a receipt on whether users’ efforts to perform different activities were sufficient for reaching the activity goals. Needless to say, this requires that the measurements of different activities are reliable and that the devices must be robust in their functioning. Additionally, it is important that the goal set is reasonable for the individual. This indicates that the used AMs could support the users own exploring of PA behavior. Therefore, we believe that the AMs shall support the BCT “model or demonstrate the behavior.” In this respect, we see room for improvements of the devices to strengthen their support for this BCT. Additionally, the AM app could be enriched with features supporting the BCT “barrier identification or problem solving,” which could be critical for individuals that for different reasons were hindered in exploring PA behavior.

Users’ experiences about being monitored include valuable information about preferences for support, as well as preferred key values of the technical devices. Here, the users described their need for feedback on accomplished PA as a positive receipt on their efforts during the day. Additionally, they liked to be praised for having been active. Hence, this illustrated the importance of the BCT “rewards contingent on successful behavior” for seniors striving toward increasing their PA. High reliability of the measurements is a necessary prerequisite such that users would perceive them useful and meaningful. Moreover, using the wearable must be perceived as smooth, comfortable, and nondemanding for the users to accept the devices in a long-term perspective.

Finally, users’ experiences related to handling the technology contains valuable information on user requirements related to usability. Here, the users stressed that the devices must be easy and robust to use. They argued that the technology should be comprehensible, intuitive, and self-instructive to prevent users feeling insecure on the handling.

### Limitations

These results cannot be generalized to all community-living older adults as further described below. Hence, the following limitations of the study have been identified.

### Participants

The number of participants was few but considered as sufficient because of the relative homogeneity of the group [40]. The analysis of the interviews indicates that saturation in terms of emerging categories was obtained. Moreover, all recruited participants were positive toward technical support and/or fall prevention training. Hence, for studying experience of persons reluctant to using technology or being physically active, this group of participants might not be representative. Analysis of the activity diaries showed large variance in self-estimated daily physical effort between the participants. Additionally, self-estimated daily effort varied over time for each person. This can be explained by the participants’ different physical conditions. Both individuals who were users and nonusers of walking aids were included.

### Activity Monitors and Tablet-Based Apps

Only two different monitors were tested in the group; both of them were used together with a corresponding specific app on a tablet. User experience may vary over time and by model of monitor. However, the inclusion of two different products makes our results not just representative for one specific AM.

### Intervention

Participants tested the technology for 9 to 10 days. Experience and acceptance vary over time and the period used is short. However, the used length of testing period enabled us to identify key challenges and experiences by new users in the critical initiation phase. Recommendations on aspects related to long-term usage were deduced from the users’ perceptions of the short-term usage.

### Comparison With Prior Work

Persuasive technology is designed to support behavioral change [11]. In our study, the participants described that the technology increased their awareness of how active they actually were. Increased self-awareness of PA has also been described in other studies of older adults’ acceptance of wrist worn AMs [27]. However, the technology’s influence on motivation and behavioral change related to PA varied between participants in our study. The ability of AMs to provide more awareness than motivation in PA with goal setting and progress monitoring has been demonstrated in other studies with younger users [41]. In addition, it has been suggested that current AMs need to be enriched with additional BCTs that are more likely to appeal to senior users [23]. French and coworkers have identified “provide rewards contingent on successful behavior,” “barrier identification or problem solving,” and “model or demonstrate the behavior” as the most effective for increasing older adults’ PA [22].

In our study, some of the participants were already motivated for being physically active and had already included regular PA in their daily lives. At least partly, this could be referred to as sample selection bias as the participants had already shown some interest for PA. These persons had their own views on adequate activity behavior and own personal aims. Furthermore, if hinders had occurred to them, they had been able to manage them. Meanwhile, other participants had low motivation for being physically active, something that was not affected by the monitors. It is possible that some of these participants did not perceive the technology as motivational. Possible reasons for this are that the BCTs incorporated in the products were insufficient for supporting and motivating those persons [9,22,23]. For example, barrier identification or problem solving might be highly valuable for persons experiencing different kind of hinders for PA. Other explanations can be poor usability and insufficient comprehensibility of the devices. For example, several participants perceived the provided feedback as difficult to interpret and value. Moreover, some participants had difficulties in handling the devices and therefore missed out on the motivating feedback. This confirms the information processing theory [42] describing that a person must both receive and comprehend the persuasive message to be able to change attitude. In addition, participants identified users’ personal interest in and motivation for progress as a prerequisite for using AMs as support for PA. Patel has similarly pointed out this opinion [39]. This indicates that for persons with low PA levels, low motivation, and low interest in progress, current AMs might not be suitable. For them, new persuasive solutions meeting their needs for motivation are necessary.

The emotions expressed in relation to the technology were described as enjoyable, positively surprising, but also embarrassing if it was related to feedback of low PA. Some participants perceived PA as funnier when being monitored. Positive emotions are important to notice as emotional meaning is prioritized and valued as more relevant than instrumental gains in the old population. The emotional meaning is also closely related to motivation in this age group [43]. Negative emotions expressed were connected to disappointment when the measurements failed. In this respect, the importance of the technology’s reliability was emphasized. This opinion relates to theoretical models of technology adoption [44] and efficient persuasive technology [11]. A systematic review of older adults’ perception of technologies aimed at fall prevention, detection, and monitoring has identified that the technology must be simple, reliable, effective, and tailored to individual need [45].

Our study adds new knowledge to prior work on older people’s experiences in using AMs. Recently, a quantitative study measured older users’ experiences of commercial AMs for self-monitoring of PA in terms of drivers for technology use from TAM [25]. It has been suggested that additional variables should be included in TAM for better reflecting older people’s technology acceptance behavior [26]. Our qualitative methodology enabled us to identify motivational aspects as highly relevant. Here, we found that the measurements’ impact on motivation for PA varied between participants.

Moreover, our study has applied a different setup during the technology intervention compared with the study by McMahon and coworkers [25]: the technology used in their study comprised one type of activity bracelet (without tablet) that was used for a significantly longer period of time together with extensive access to support for the users. Our study confirms that older adults perceive activity bracelets easy to use. Moreover, the tablet was perceived difficult to maneuver by our participants who had a median age of 83 years. This confirms previous studies reporting that using tablets among individuals older than 60 years can be associated with problems [46]. As support, our participants had received written information on how to handle the technology. Despite this, they realized that they would have needed more support for learning the handling. In earlier studies, it has been highlighted that older adults need support through the process of learning how to use new technology [47]. We now realize that our participants needed more supported learning time, even if this was not requested when the participants met the physiotherapist. In this respect, we believe that our study setting is closer to the real-life situation of a senior person starting to use any commercially available AM as support for PA.

Furthermore, mixed-methods evaluations of usability, usefulness, and acceptance of wearable AMs for adults over 50 years with chronic illness [28] and community-dwelling adults between 55 and 84 years have been performed [27]. In the study by Mercer [28], users with chronic illness tested five AMs for 3 days and evaluated the devices’ usability and usefulness by questionnaires based on TAM. Moreover, qualitative data was collected in focus groups and subjected to thematic analysis. Despite differences in age and health status between these users and the participants in our study, similarities in the users’ experiences can be identified: both groups described that using the AMs increased awareness of their PA levels. However, the users with chronic illness [28] had already been asked by a physician to exercise more. Hence, the increased self-awareness contributed increased motivation for PA. When aiming at increasing peoples PA for preventing future disease, the potential risks because of current behavior and the potential benefits gained through altered behavior need to be perceived by the user.

In the study by Puri [27], the users tested two different AMs for 3 weeks. A questionnaire gathered users’ experience and acceptance after each testing period. The users expressed moderate levels of acceptance. In addition, semistructured interviews were conducted with 4 participants and analyzed with regards to qualitative content. Here too, participants described that the AMs had increased their self-awareness and motivation for behavioral change. The AM’s impact on motivation for behavioral change varied among participants in our study.

An unexpected finding was that the participants in our study did not experience any problem related to integrity when using the technology. In fact, usually privacy concerns are significantly associated with wearable technology acceptance in health care in the general population [48]. However, the view of the participants in our study regarding integrity has also been described in the study performed by Puri [27] and in reviews of studies on ethical considerations concerning assistive technology [49]: the majority of older people state that the needs for devices overrule any possible privacy concerns, and as long as there is a balance between needs and privacy, they do not feel that their privacy is violated. This opinion could possibly also reflect that the users, who had limited previous experience of using the Internet, had limited knowledge and awareness of integrity aspects related to recording of PA. This question needs to be addressed in future studies.

### Conclusions

The study investigated senior users’ experience in using AMs as support for PA in daily life. Conclusions to be drawn from the study are as follows:

- AMs can increase senior users’ awareness of own PA behavior.
- The influence of AMs on older users’ motivation and/or PA behavior varies between different senior users: although some users started to explore how different activity behavior affected PA levels, other persons maintained their daily PA habits.
- For the measurements to be perceived meaningful and useful for the users, they have to be reliable and give the user a receipt on whether the daily PA has been enough in relation to a quantitative goal. Feedback in terms of praise is also appreciated.
- For AMs to be useful in the long-term for senior users, the devices must be easy to use, intuitive, robust, and reliable. Deficiencies in these areas significantly reduce the users' motivation in using the AMs.
- Current AMs partly support BCTs effective for increasing older adults’ PA. However, the devices should be further developed and enriched to better support effective BCT for the target group.
- Participants in the study expressed no problems related to integrity when using the AMs. Whether this experience reflects limited awareness of integrity issues related to Internet-based registration of PA needs to be addressed in future studies.

In summary, this study has provided insights on how senior community-living adults with little or no experience of information and communication technology perceived using AMs. AMs can be valuable for supporting some older adults’ PA. However, currently available products are not ideal for broader groups of older users.

## Appendix

Multimedia Appendix 1 Interview guide.

Multimedia Appendix 2 Main and subcategories based on the interviews.

## Abbreviations

AM: activity monitor
BCT: behavior change technique
PA: physical activity
TAM: technology acceptance model
