# Supplementary material for: Activity Monitors as Support for Older Persons’ Physical Activity in Daily Life: Qualitative Study of the Users’ Experiences
Source: JMIR Mhealth Uhealth. 2018 Feb 1;6(2):e34. doi: 10.2196/mhealth.8345 (PMC5814603; doi:10.2196/mhealth.8345)
Supplement: Multimedia Appendix 1 [file mhealth_v6i2e34_app1.pdf]

Multimedia Appendix 1 Interview guide

1. *Main question:* What are your thoughts and experiences of using the technical devices during the testing period? What is your overall, general experience? What have you learnt?
   Follow-up questions were adjusted to responses of main questions.
   Example of follow-up questions:
   Did you experience any inconvenience caused by the technology during the testing period?

2. *Main question:* What aspects are important for a technical support that you would like to use?
   Follow-up questions were adjusted to responses of main questions and focused on motivation, motivating elements, integration of training in daily routines, support for goal-setting, usability and integrity
   Example of follow-up questions:
   How could a technical support motivate you to increase your physical activity?
   What do you think of the devices usability?
   You describe that the technical devices must be easy to use; can you give examples of how?
   Would you like to describe what you think must be changed with these devices?
   How do you look upon aspects like integrity in relation to measurements of physical activity?

3. *Main question:* What is your experience of seeing the activity results on the screen? How did you feel? What did you think? How would you have preferred to get feedback on your activity results?
   Follow up questions using ”How” were used in order to explore the users’ experiences of studying the activity results on the screen.
   Based on the users’ descriptions, further follow-up questions regarding the users’ preferences on feedback were asked.
   Example of follow-up questions:
   How do you perceive that feedback from the technical support could motivate you to increase your physical activity?
   You mentioned that the information should be clear, could you give examples of how?
