# Supplementary material for: Activity Monitors as Support for Older Persons’ Physical Activity in Daily Life: Qualitative Study of the Users’ Experiences
Source: JMIR Mhealth Uhealth. 2018 Feb 1;6(2):e34. doi: 10.2196/mhealth.8345 (PMC5814603; doi:10.2196/mhealth.8345)
Supplement: Multimedia Appendix 2 [file mhealth_v6i2e34_app2.pdf]

Multimedia Appendix 2 Main – and subcategories based on the interviews

Main category	Adherent sub-categories
Influence on the individual	Motivation
	Emotions
	Awareness
	Behavior
Experiences from being monitored	Limitations
	Possibilities
	Integrity
	Reliability
	Feedback
Experiences from using the technical devices	Handling
	Learning
	Insecurity
	Wearing the monitors
